# Effect of Whitening Toothpastes with Different Active Agents on the Abrasive Wear of Dentin Following Tooth Brushing Simulation

**DOI:** 10.3390/jfb14050268

**Published:** 2023-05-12

**Authors:** Dimitrios Dionysopoulos, Spyros Papageorgiou, Constantinos Papadopoulos, Sotiria Davidopoulou, Avraam Konstantinidis, Kosmas Tolidis

**Affiliations:** 1 Department of Operative Dentistry, School of Dentistry, Aristotle University of Thessaloniki, 54124 Thessaloniki, Greece; cospapadopoulos@hotmail.com (C.P.); sdavidop@dent.auth.gr (S.D.); ktolidis@dent.auth.gr (K.T.); 2 Laboratory of Chemistry-Biochemistry-Cosmetic Science, Department of Biomedical Sciences, University of West Attica, Panepistimioupolis Egaleo Park, 12243 Athens, Greece; spapage@uniwa.gr; 3 Department of Civil Engineering, Division of Structural Engineering, Faculty of Engineering, Aristotle University of Thessaloniki, 54124 Thessaloniki, Greece; akonsta@civil.auth.gr

**Keywords:** abrasive wear, charcoal, dentin, surface height parameters, surface loss, whitening toothpastes

## Abstract

The aim of this research was to evaluate the abrasive dentin wear that can be induced by three commercial whitening toothpastes following a tooth-brushing simulation (TBS) corresponding to a three-month period. Sixty human canines were selected, and the roots were separated from the crowns. Then the roots were randomly divided into six groups (*n* = 10) and were submitted to TBS using the following slurries: Group 1—deionized water (RDA = 5); Group 2—ISO dentifrice slurry (RDA = 100); Group 3—a regular toothpaste (RDA = 70); Group 4—a charcoal-containing whitening toothpaste; Group 5—a whitening toothpaste containing blue covasorb and hydrated silica; and Group 6—a whitening toothpaste containing microsilica. Following TBS, surface loss and surface roughness changes were evaluated using confocal microscopy. Additionally, surface morphology and mineral content changes were observed using scanning electron microscopy and energy-dispersive X-ray spectroscopy. The deionized water group presented the lowest surface loss (*p* < 0.05), while the charcoal-containing toothpaste presented the highest surface loss, followed by ISO dentifrice slurry (*p* < 0.001). Blue-covasorb-containing and regular toothpastes did not present statistically significant differences (*p* = 0.245), and neither didmicrosilica-containing toothpaste or ISO dentifrice slurry (*p* = 0.112). The surface height parameters and surface morphology changes of the experimental groups followed the surface loss patterns, while no differences were detected in mineral content after TBS.Although the charcoal-containing toothpaste exhibited the highest abrasive wear to dentin, according to ISO 11609, all the tested toothpastes exhibited appropriate abrasive behavior towards dentin.

## 1. Introduction

Whitening toothpastes are a type of “over-the-counter” product commonly used for teeth whitening. As the demand for optimal tooth aesthetics increases, various products have been introduced promising whitening effects within 2–6 weeks as an alternative to at-home or in-office tooth bleaching treatments [1,2]. Unlike bleaching gels that release high amounts of hydrogen peroxide (H_2_O_2_) and modify the chromophores of tooth tissues during in-office or at-home bleaching treatments, whitening toothpastes involve multiple mechanisms of whitening action [3]. These may include the following: (a) abrasive particles (hydrated silica, perlite, alumina, calcium carbonate, calcium pyrophosphate, sodium bicarbonate, etc.) that remove extrinsic stains and dental plaque via frictional forces; (b) chemical substances (hydrogen peroxide, calcium peroxide, sodium citrate, sodium pyrophosphate, papain, etc.) that chemically interact with the chromophores of tooth tissues by breaking down their molecules and changing their size, geometry, and polarity to alter the color of the teeth [4]; and (c) optical brighteners such as blue covarine or blue covasorb, which are actually dyes that cover the surfaces of the teeth and enhance tooth whiteness perceptibility [2].

Abrasive particles are included in almost all toothpastes available in the market that assist to remove microbial plaque and extrinsic stains without inducing significant damage to tooth and gingival tissues. Whitening toothpastes typically contain higher amounts of abrasive particles than regular toothpastes, as they aim to achieve an improved whitening effect by removing a higher number of extrinsic stains [5]. As a consequence, the risk for abrasive dentin wear is higher for whitening toothpastes and it has been postulated that they constitute a crucial factor in the development of noncarious cervical lesions [6] and gingival recession [7]. Tooth surface loss may induce dentin hypersensitivity [8], tooth color change [9], and lower mechanical strength of the teeth [10]. Furthermore, surface roughness of the teeth may be increased, leading to the accumulation of larger amounts of biofilms and, thus, to a higher risk of caries formation and periodontal inflammation [11]. For this reason, whitening toothpastes should be administered with cautiousness or avoided in patients susceptible to erosive tooth wear.

A more recently introduced category of whitening dental products includes activated-charcoal-containing toothpastes, which potentially offer detoxifying benefits and antiseptic or antifungal activity, albeit there is no clinical evidence for this behavior in clinical conditions [12]. Activated charcoal is a type of carbon that has been processed to have a nanocrystalline structure, providing it with a high surface area and porosity in the nanometer scale. This unique structure makes it an effective adsorbent for a wide range of applications [13]. Activated charcoal functions by adhering to deposits on the surface of teeth, such as extrinsic stains or microbial biofilms, and taking them into its pores. Subsequently, these deposits are removed during tooth brushing along with the charcoal [13], leading to a whiter color of the teeth [14]. The potential for abrasive wear may also exist in toothpastes that contain charcoal, which can vary depending on the type, preparation method, and size distribution of the activated charcoal particles. Notwithstanding that abrasivity of toothpaste is necessary for its effectiveness in removing extrinsic chromophores, resulting in tooth whitening, high abrasivity or extensive use of such toothpastes may cause surface loss and changes in surface roughness and the morphology of the dentin exposed to tooth brushing [15].

Quantitative evaluation of abrasive dentin wear after brushing with whitening toothpastes can be implemented by measuring the amount of dentinal tissue that was removed. The standard approach involves assessing the relative dentin abrasivity (RDA) of the toothpaste, which can be measured using either the radiotracer (Rt) method or the profilometry equivalent method (RDA-PE) [16] in accordance with the guidelines set out in ISO 11609:2017(E) [17]. Nevertheless, the complexity of the method and the time-consuming and cost-intensive procedure, as well as its sensitivity to various factors such as dentinal substrate and reference abrasives, have led to modifications of this testing. Additionally, qualitative evaluation of abrasive dentin wear is useful for investigating the mechanisms of the degradation of dentin structure during tooth brushing. In particular, surface roughness and morphology alterations can be considered for the interpretation of the results [18].

Therefore, the aim of the present research was to evaluate the abrasive dentin wear that can be induced by three commercial whitening toothpastes with different active agents (activated charcoal, blue covasorb, and microsilica) and to compare it with that induced by a regular toothpaste (hydrated silica) with medium abrasivity (RDA: 70), following a tooth-brushing simulation corresponding to a three-month period. Additionally, changes in the surface roughness and morphology of the brushed dentin were observed to qualitatively evaluate abrasive wear using confocal microscopy and scanning electron microscopy (SEM). The RDA of the tested whitening toothpastes was estimated using a modified profilometry equivalent method (RDA-PE). The first null hypothesis (H_0_1) of the study was that the tested whitening toothpastes would induce the same surface loss of dentin after the tooth-brushing simulation. The second null hypothesis (H_0_2) of the study was that the tested whitening toothpastes would similarly change the surface roughness of the dentin after the tooth-brushing simulation.

## 2. Materials and Methods

### 2.1. Preparation of the Dentin Specimens

A total of sixty undamaged human canine teeth were extracted due to periodontal issues and preserved in a 0.5% chloramine T solution at a temperature of 6 °C until required, with a maximum storage period of three months. The age of the patients was in the range of 5 years and they gave their consent to use their teeth for research after they were informed of the purpose of this investigation. After the extraction of the teeth, the remaining soft tissues were carefully removed and the tooth surfaces were cleaned with slurry of pumice and water. Then the roots were separated from the crowns using a water-cooled diamond disc (Isomet, Buehler, Lake Bluff, IL, USA) and stored in artificial saliva at 37 ± 1 °C. The composition of the artificial saliva was as follows: 0.103 g/L of CaCl_2_, 0.019 g/L MgCl_2_•6H2O, 0.544 g/L KH_2_PO_4_, and 2.24 g/LKCl; and buffer (TCP-KOH) was added to adjust the pH to 7 [19]. The roots of the teeth were placed with their labial surfaces facing upwards into self-cured acrylic resin (NT Newton, Toros Dental, Antalya, Turkey), after which the dentinal surfaces were polished using a grinding machine (Jean Wirtz TG 250, Dusseldorf, Germany) with a water-cooling system (50 mL/min) at a rotational speed of 200 rpm. The polishing process involved using silicon carbide abrasive papers (Apex S system, Buehler, Lake Bluff, IL, USA) with gradually increasing grit sizes of 600, 800, 1000, and 1200. Subsequently, the specimens were immersed in ultrasonic bath (Euronda Spa, Montecchio Precalcino, Vicenza, Italy) for 5 min and were stored in artificial saliva at 37 ± 1 °C until used.

### 2.2. Experimental Groups of the Study

The root specimens were randomly divided into 6 groups (*n* =10) and were submitted to tooth-brushing simulation (TBS) using the following slurries:

Group 1 (negative control group): No toothpaste was used during TBS, only deionized water (RDA = 5).
Group 2 (positive control group): Freshly prepared ISO dentifrice slurry was used during TBS, which corresponds to RDA = 100. To prepare it, 10 g of calcium pyrophosphate was added to 50 mL of the reference diluent, which comprised 100 mL of glycerin, 5 g of carboxymethyl cellulose, and 900 mL of deionized water. The mixture was then stirred at a slow pace overnight at a temperature of 23 °C [17].
Group 3: A regular toothpaste (Colgate Total^®^, Colgate-Palmolive Company, Greece) with medium relative dentin abrasivity (RDA = 70) was used during TBS.
Group 4: A charcoal-containing whitening toothpaste (Black & Polish Toothpaste, Frezyderm, Greece) was used during TBS.
Group 5: A whitening toothpaste containing blue covasorb and hydrated silica as active agents (Instant Whitening Blue Toothpaste, Frezyderm, Greece) was used during TBS.
Group 6: A whitening toothpaste containing microsilica as active agent (Whitening Toothpaste, Frezyderm, Greece) was used during TBS.

The slurries were made by diluting 0.2 g of the tested toothpaste in 500 µL of distilled water, which was applied directly on the surface of dentin with a plastic syringe. The compositions of the tested commercial toothpastes are presented in Table 1.

### 2.3. Tooth-Brushing Simulation (TBS)

**Figure 1:**
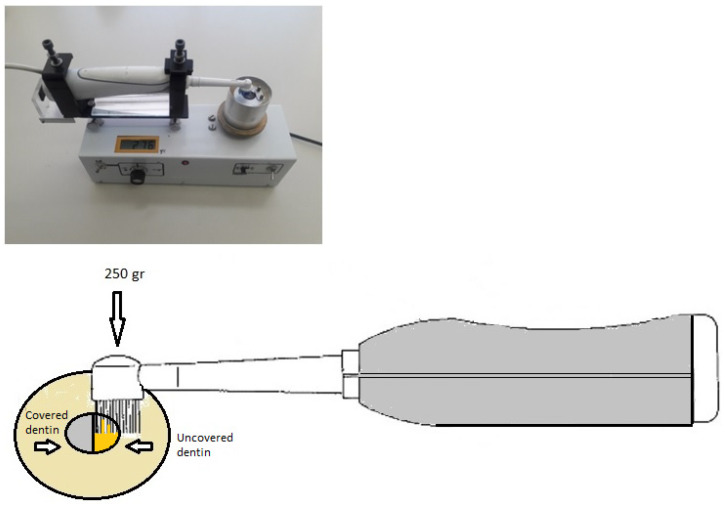
The constructed apparatus that was used to fix the electric toothbrush to a standard position for tooth-brushing simulation. The set-up of the electric toothbrush with the specimen is illustrated in the lower image.

For the purpose of simulating tooth brushing, a commercial electric toothbrush (Oral-B, Braun, France) was employed in the study. The toothbrush was set to specific parameters, including a standardized load of 250 g (≈2.45 N), a medium-hardness toothbrush head (Oral-B EB20, Procter & Gamble, Athens, Greece), a rotation rate of 7500 rpm, and a change in rotation direction every 30 s. The toothbrush head was replaced after every 5 specimens, with each group (*n* = 10) being brushed with two new toothbrush heads. An apparatus was designed to secure the electric toothbrush in place and ensure that the toothbrush head remained parallel to the dentin surface, with the applied pressure being monitored with an electric scale (Figure 1). The toothbrush head was in contact with the dentin specimen constantly during TBS and the slurries were prepared just before each brushing cycle and with vigorous stirring. Taking into consideration that most individuals brush their teeth twice a day for an average of 2 min, and that there are a total of four quadrants with three tooth surfaces each to be cleaned, with the length of a toothbrush being able to cover only two teeth at a time, it is reasonable to set a 10s brushing time for each tooth surface as the representative time for one person’s daily tooth brushing. To simulate 3 months of daily tooth brushing, the duration of the brushing procedure for each specimen was set at 15 min. Following the brushing procedure, the specimens were rinsed with deionized water for 20 s, air-dried for 5 s, and then submerged in artificial saliva at 37 °C.

### 2.4. Evaluation of Surface Loss of Dentin

**Figure 2:**
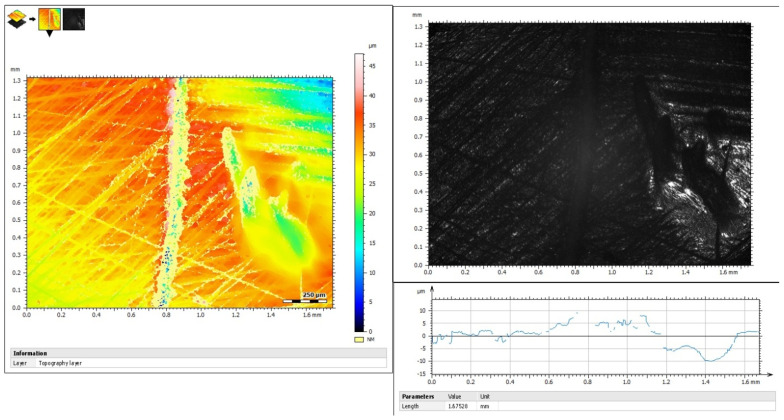
Representative image of a dentin specimen after tooth-brushing simulation. The left part is the baseline surface and the right part is the brushed surface. In the middle, there is the groove that separates the covered and uncovered surfaces from the adhesive tape.

Before TBS procedure, half of the dentin’s surface of each specimen was covered with one-sided silver adhesive tape (Wonder^®^ Tape, Achem Technologies, Taipei City, Taiwan) as is shown in Figure 1. To maintain the reference surfaces and ensure accurate measurement of abrasion depth, a groove was marked with a surgical blade in the middle of the surface at the limit with the adhesive tape. After the brushing simulation, surface loss was evaluated using a confocal microscope (3D Optical Surface Metrology System Leica DCM8, Leica Microsystems CMS GmbH, Mannheim, Germany) at interferometry mode (brightfield), with a green LED irradiating at 530 nm. Four images were taken at different areas of the center of each specimen’s surface, corresponding to a surface area of 1.31 × 1.72 mm^2^ for each image. Surface loss was measured by superimposing the baseline and post-treatment profiles, and abrasion depth was calculated by subtracting the two profiles. Five measurements were taken in each image, 40 μm apart, and the data were averaged and reported in μm. An example of an image used for evaluating surface loss can be seen in Figure 2.

### 2.5. Evaluation of Surface Height Parameters of Dentin

**Figure 3:**
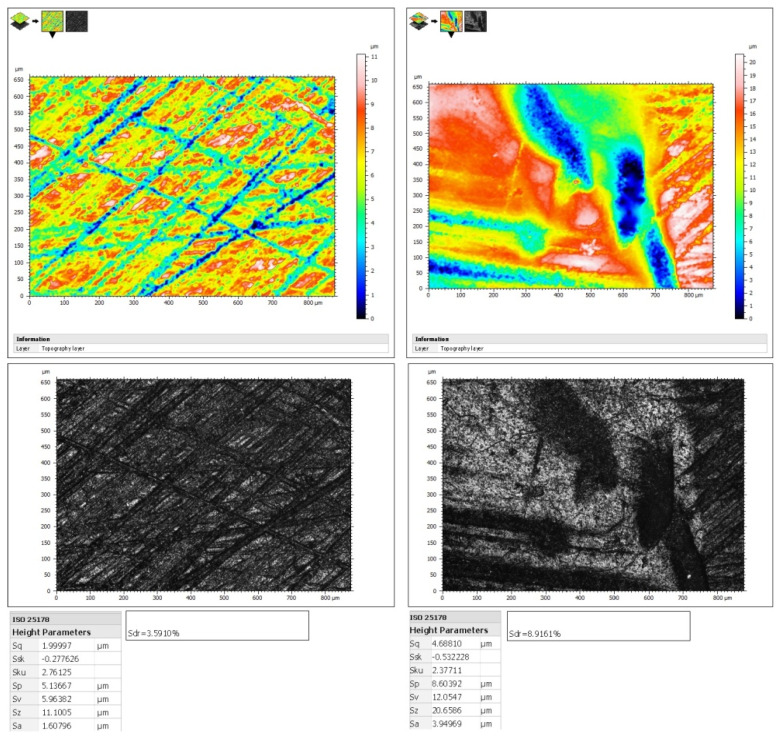
Representative images of covered (right) and uncovered (left) dentin after tooth-brushing simulation. The height and hybrid (Sdr) parameters of each surface according to ISO 25178, which were calculated with the software of the confocal microscope, are presented below the images.

Surface height parameters of dentin were evaluated following ISO 25178 (noncontact type) [20] using confocal microscopy at optical profilometer mode. Four images were captured from the center of each quadrant of both the brushed and unbrushed surfaces of each specimen, resulting in a surface area of 0.880 × 0.660 mm^2^ for each image (magnification ×20). Leica Map software (Leica GmbH, Germany) was utilized to obtain data and determine the arithmetical mean height of the dentin surface in Sa (μm), the maximum average between the highest peaks and valleys of the surface in Sz (μm), and the mean developed interfacial area ratio in Sdr (%). The developed interfacial area ratio is a measure of the ratio of the apparent surface to the actual surface of a material. Representative images of the covered and uncovered dentin surfaces used to evaluate their surface properties are depicted in Figure 3.

### 2.6. SEM and EDS Surface Analysis of Dentin

Scanning electron microscopy (JEOL Ltd., JSM-840, Tokyo, Japan) was utilized to examine morphological changes between intact and brushed surfaces in three dentin specimens (*n* = 3) of each experimental group. The specimens were mounted on aluminum stubs, sputter-coated with carbon to a thickness of about 200 Å in a vacuum evaporator at low vacuum, and observed at an accelerated voltage of 20 kV and a working distance of 9 mm. Photomicrographs were taken from the brushed surfaces at ×100, ×500, and ×1000 magnifications to evaluate morphological changes. Energy-dispersive X-ray spectroscopy (EDS) was also employed to assess differences in mineral content of dentin after TBS among the experimental groups.

### 2.7. Statistical Analysis

The statistical analysis of the results was conducted using IBM Corp’s SPSS Statistics 25.0 software (Chicago, IL, USA). The sample size was determined to detect a minimum of 60% difference between any two groups with a power of 80% at a significance level of 5%. Normality and homogeneity of the data were checked using the Shapiro–Wilk and Levene tests, respectively. Surface loss and surface parameter data were subjected to one-way ANOVA, and Tukey’s test was used to determine significant differences between the experimental groups at a significance level of 0.05.

## 3. Results

### 3.1. Surface Loss Outcomes after TBS

The means and standard deviations of surface loss in μm of the experimental groups of the study after TBS are presented in Table 2. Tooth-brushing simulation induced surface loss in all the experimental groups, but to different extents. More specifically, the negative control group (deionized water) presented the lowest surface loss, as was expected (*p* < 0.05), while the positive control group (ISO dentifrice slurry) presented the second-highest surface loss, following Black & Polish Toothpaste, which induced the highest surface loss (*p* < 0.001). Instant Whitening Blue Toothpaste and Colgate Total did not present statistically significant differences (*p* = 0.245), and neither did Whitening Toothpaste or the positive control group (*p* = 0.112). This section may be divided by subheadings. It should provide a concise and precise description of the experimental results, their interpretation, and the experimental conclusions that can be drawn.

**Figure 4:**
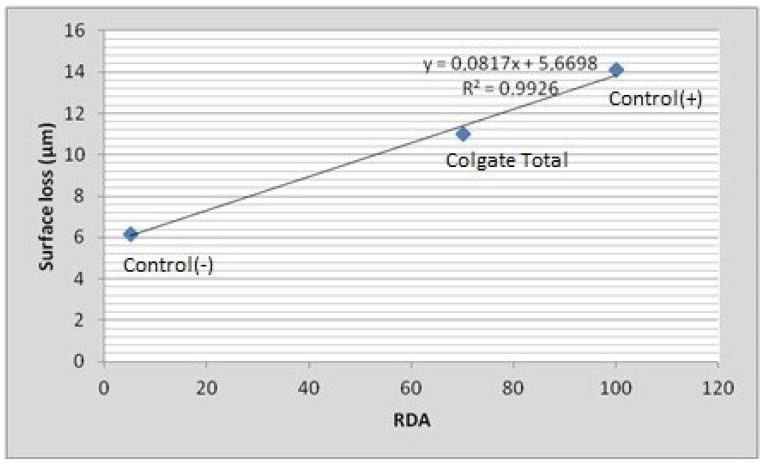
Linear regression analysis between surface loss (μm) after tooth-brushing simulation and relative dentin abrasivity (RDA) of the tested slurries with known RDA. The coefficient of determination (R^2^) indicates a strong correlation between surface loss and RDA.

Calculation of the RDA-PE for the tested dentifrice slurries (Table 2) was implemented using the equation y = 0.0817x + 5.6698, which was derived by the linear regression analysis between the surface loss and RDA of the slurries with a known RDA (Figure 4). The coefficient of determination (R^2^) was 0.9926, indicating a strong correlation between surface loss and RDA. According to ISO 11609, the abrasivity of a dentifrice slurry is low when RDA = 0–70, medium when RDA = 71–100, and high when RDA = 101–150. As a result, Instant Whitening Blue Toothpaste has low abrasivity, Whitening Toothpaste has mediumabrasivity, and Black & Polish Toothpaste has highabrasivity.

### 3.2. Surface Height Parameter Changes after TBS

The means and standard deviations of the arithmetical mean height (Sa), maximum average between the highest peaks and highest valleys of the surface (Sz), and mean developed interfacial area ratio (Sdr) of the experimental groups of the study before and after TBS are shown in Table 3, Table 4 and Table 5, respectively.

All the tested dentifrice slurries induced an increase in Sa values after TBS but to different extents (*p* < 0.05), from a slight (11.01%) to a remarkably high (145.64%) rise. The highest increase in Sa was exhibited bythe charcoal-containing dentifrice (Black & Polish Toothpaste) with an almost double increase compared withthe positive control group which followed (*p* < 0.001). The lowest increase in Sa after TBS was presented by Whitening Toothpaste together with the negative control, which did not differ significantly (*p* > 0.05). Colgate Total and Instant Whitening Blue Toothpaste presented moderate increases in Sa following TBS (Table 3).

Regarding Sz values, the highest increase was also exhibited by Black & Polish Toothpaste (*p <* 0.001), followed by the positive control group and then Instant Whitening Blue Toothpaste (*p <* 0.05). The other three groups presented slighter increases with no significant differences from each other (*p* > 0.05) (Table 4).

Following TBS, all the experimental groups presented increases in Sdr values (*p* < 0.05), but to similar extents, ranging between 20.23 and 33.21%, except for Black & Polish Toothpaste, which exhibited a significantly higher increase (59.39%), as can be seen in Table 5.

### 3.3. Surface Morphology Alterations after TBS

**Figure 5:**
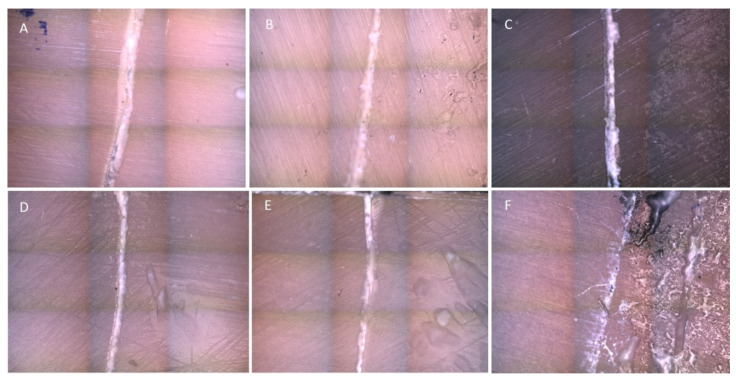
Representative images of the experimental groups of the study showing morphological differences between brushed (right) and covered (left) surfaces of dentin using confocal microscopy (magnification×20). (**A**): deionized water; (**B**): Instant Whitening Blue Toothpaste; (**C**): Colgate Total; (**D**): Whitening Toothpaste; (**E**): ISO dentifrice slurry; (**F**): Black & Polish Toothpaste.

**Figure 6:**
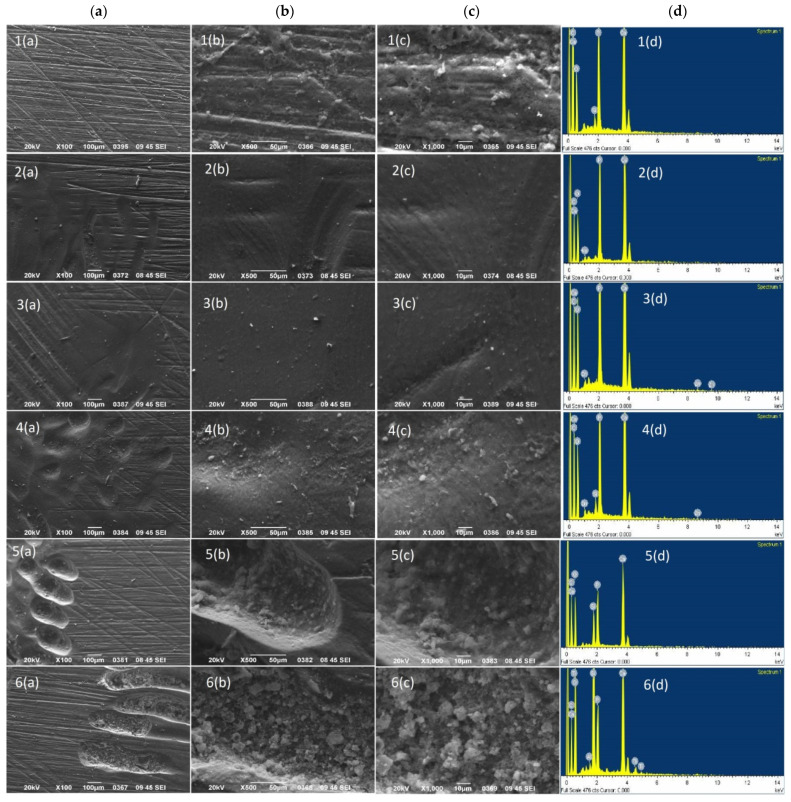
Representative SEM images of the experimental groups of the study showing morphological alterations of the brushed surface of dentin at three magnifications: ×100 (**a**), ×500 (**b**), and ×1000 (**c**). The EDS spectra of the images at the right (**d**) indicate mineral content of the brushed surfaces. Image1: deionized water; Image2: Instant Whitening Blue Toothpaste; Image3: Colgate Total; Image4: Whitening Toothpaste; Image5: ISO dentifrice slurry; Image6: Black & Polish Toothpaste.

Representative images of the samples of each experimental group showing covered and uncovered dentin after TBS using confocal microscopy (magnification ×20) are illustrated in Figure 5. Additionally, representative SEM images of the brushed surfaces of the dentin specimens of each experimental group at three magnifications (×100, ×500, and ×1000), accompanied with the respective EDS spectra, are shown in Figure 6. Alterations in surface morphology followed the outcomes of the surface loss of the experimental groups. Defects such as craters and scratches were observed at the areas where the toothbrush was applied. Black & Polish Toothpaste and the positive control presented the largest and deepest defects, followed by Whitening Toothpaste, which showed less intense surface features and irregularities, while in the Colgate Total and Instant Whitening Blue Toothpaste groups only minor alterations on the surface were observed. In the negative control group, the surface appeared uniform and smoother with negligible changes compared with the covered surface. EDS analysis of the surfaces did not reveal any significant differences in mineral content among the groups of the brushed surfaces (*p* > 0.05) (Table 6).

## 4. Discussion

The primary factors that are believed to impact the abrasion of dentin caused by toothpaste are the abrasive agents contained in the toothpaste, the pressure applied during brushing, the duration of brushing, and the bristle stiffness of the toothbrush [21,22]. In the current research, we focused on the abrasive ability of the toothpastes. To evaluate the impact of a toothpaste’s abrasiveness on tooth surfaces, it is important to analyze both surface loss and surface roughness. This provides insights into the extent of the tooth substance that is worn away and the roughness of the surface that remains [18]. Studies indicate that abrasiveness tends to rise as particle size increases until the particles become too big to be contained by the bristles and are instead pushed aside by the toothbrush. The noticeable range in the average depth of abrasion seen across various dentifrice slurries tested could be due to differences in particle size distribution and composition, as well as variations in the dentin of individual canine teeth [23].

Based on the results of the current study, the first null hypothesis (H_0_1), which stated that the tested whitening toothpastes would induce the same surface loss of dentin after the tooth-brushing simulation, was rejected. According to the findings of the present study, the toothpaste containing charcoal resulted in the greatest loss of dentin surface. This is in line with a recent review article on charcoal-based toothpastes, which noted that only 28% of such products have low abrasiveness [12]. Toothpaste’s abrasive action on tooth surfaces is influenced by various factors, such as the hardness, shape, size, distribution, and concentration of abrasive particles [24]. The abrasive components of whitening toothpastes must promote therapeutic effects without harming tooth hard tissues, leading to better cleaning and decreased tooth wear [2]. It has been assumed that the abrasive power of toothpastes containing charcoal relies on the type of charcoal used, the method of preparation, and the particle size distribution [15]. Earlier studies showed that oral hygiene products containing charcoal have relatively high abrasiveness [25]. This is in line with the results of the current investigation.

Regarding the effectiveness of whitening toothpastes, a study found that a toothpaste containing activated charcoal was more effective at whitening teeth compared with a regular toothpaste used as the control group. However, it was found to be less effective than other toothpastes that contained whitening agents such as microbeads, hydrogen peroxide, or blue covarine [13]. A more recent study also reported a higher whitening effect for the same charcoal-containing toothpaste that was used in the current study compared with a regular toothpaste (Colgate Total) [14]. This may be due to its higher abrasivity, as was found in the current study. However, a recent review of the literature concluded that there is inadequate evidence to support the claim that toothpastes containing charcoal offer a better whitening effect [12].

As previously stated, activated charcoal’s capacity to soak up external tooth stains within its pores and then be brushed off results in tooth whitening. However, there is limited scientific proof to support this claim, and it is generally believed that charcoal only alters tooth color through abrasive action similar to that of conventional toothpaste. Numerous studies have established that toothpaste with a higher level of abrasiveness is more effective in terms of cleaning and whitening teeth [12]. However, it also has a downside as it causes faster and more significant tooth wear [26]. To assess the safety of these products for daily use, a more thorough examination of surface roughness and loss would be beneficial. The level of abrasiveness in a toothpaste is determined by its RDA (relative dentin abrasivity) units. According to ISO 11609 standards, toothpaste with an RDA of up to 150 is considered safe for proper tooth brushing. In the present study, all the tested whitening toothpastes presented RDA < 150, meaning that they can be considered safe for brushing in healthy tooth tissues.

The outcomes of the present study require rejection of H_0_2, which claimed that the tested whitening toothpastes would similarly change the surface roughness of dentin after the tooth-brushing simulation. Surface roughness is a measure of surface texture, which is determined by the deviations in the direction of the normal vector of a real surface from its ideal form [27]. Surface roughness criteria that can describe changes in tooth surfaces after TBS are Sa, Sz, and Sdr parameters, according to ISO 25178. From a clinical standpoint, surface roughness plays a crucial role. It can significantly affect the adhesion of bacteria, potentially leading to increased biofilm formation and damage to tooth hard tissues [28]. Additionally, surface roughness may contribute to gingival recession, dentin hypersensitivity, and the accumulation of extrinsic stains, all of which can impact the appearance of tooth tissues [29].

In the present study, the charcoal-containing toothpaste presented the highest surface roughness and also exhibited the highest surface loss. This aligns with Pertiwi et al.’s findings that brushing with a charcoal-containing whitening toothpaste for three months also increased surface roughness [30]. Previous research has shown that charcoal toothpaste can increase the surface roughness of dental hard tissues compared with conventional toothpaste, but this varies depending on the product [31]. Furthermore, Instant Whitening Blue Toothpaste presented a higher surface roughness increase compared with Whitening Toothpaste, albeit it presented a lower surface loss. This may be attributed to discrepancies in the shape, size, and composition of the toothpastes’particles, which induce different abrasive surfaces and different increases in the surface roughness of dentin [32]. In addition, Instant Whitening Blue Toothpaste presented a similar surface roughness increase to that of Colgate Total, while Whitening Toothpaste showed a very low increase, similar to the negative control group. These results suggest that whitening toothpastes are not necessarily more abrasive than regular toothpastes.

It is important to mention that the effects of the tested whitening toothpastes on the surface morphology of dentin can differ. In particular, the charcoal-containing toothpaste presented more intense morphological defects on the dentin after TBS than the other tested toothpastes, including deeper craters and scratches, as was revealed by SEM analysis. The findings of a recent study are in agreement with these results. The study reported that the same charcoal-containing toothpaste induced numerous large and deep craters and increased roughness on enamel, as compared with a regular toothpaste that contained hydrated silica particles. This was explained by the different properties of their abrasive agents [14].

For the TBS procedure in this study, an electric toothbrush was utilized. A previous clinical study compared the effectiveness of power and manual toothbrushes in maintaining the final color of whitened teeth, and after a six-month period, it was found that the power toothbrush group had better results in sustaining the whitening effect compared with the manual toothbrush group [33]. As a result, in the current study, an electric toothbrush was preferred.

One limitation of this study was the variability in particle size, distribution, composition, and morphology among the tested dentifrices. Additionally, because the teeth were randomly selected, there was a lack of information regarding previous conditions, such as dental age and possible interventions. The findings of this study indicate that there is a strong correlation between RDA values and absolute abrasive wear values obtained through profilometry. In contrast to previous studies, the current results demonstrate a consistent agreement between the RDA and profilometry methods [34,35].

It is worth noting that while brushing teeth regularly is important, it is also essential to use appropriate tooth-brushing techniques to avoid abrasive wear. To prevent abrasive wear, it is important to use gentle, circular brushing motions with a soft-bristled brush. Additionally, using a whitening toothpaste that is low in abrasives can help to prevent wear while still providing the desired whitening effect. By using appropriate tooth-brushing techniques and selecting the right type of toothpaste, individuals can maintain healthy and attractive smiles without compromising their tooth structure [36].

## 5. Conclusions

Within the limitations of this in vitro study, it can be concluded that the tested whitening toothpastes induced abrasive wear on dentin to different extents and showed varying morphological changes after three months of TBS. The charcoal-containing toothpaste (Black & Polish Toothpaste) exhibited the highest abrasive wear, while the Instant Whitening Blue Toothpaste containing hydrated silica and blue covasorb showed the lowest. Nevertheless, according to ISO 11609, all the tested toothpastes exhibited appropriate abrasive behavior towards dentin as they presented RDA values of less than 150. Therefore, the tested dental products could be used for tooth whitening without compromising the integrity of the dentin under the same conditions. It is important to note that the hardness of the toothbrush bristles, applied pressure, and duration of tooth brushing may be more crucial for the health of teeth and soft tissues and should be in accordance with the instructions of dental clinicians. Future studies should also evaluate the esthetic properties of the tested toothpastes and relate them with their abrasiveness.

## Figures and Tables

**Table 1:** The compositions of the tested commercial toothpastes of the study and their active agents according to manufacturers.

Product	Type	Composition	Active Agents	Manufacturer
Black & Polish Toothpaste	Whitening toothpaste	Deionized water, sorbitol, sodium saccharine, 0.32% *w*/*w* sodium fluoride (1450 ppmF^−^), sodium benzoate, polyglycol 1500s, Blanoz 7M1F Pharm, Tixosil 73, Tixosil 43, Pearlwhite 19, flavor spearmint frost EAB24297/00, biosol, citric acid, Speckare CAC3, sabosol L30	Active charcoal (1% *w*/*w*)	Frezyderm Ltd., Athens, Greece
Instant Whitening Blue Toothpaste	Whitening toothpaste	Deionized water, blue covasorb, hydrated silica, Cymenol, 0.32% *w*/*w* sodium fluoride (1450 ppmF^−^)	Blue covasorb, hydrated silica	Frezyderm Ltd., Athens, Greece
Whitening Toothpaste	Whitening toothpaste	Deionized water, microsilica, Cymenol, 0.32% *w*/*w* sodium fluoride (1450 ppmF^−^)	Microsilica	Frezyderm Ltd., Athens, Greece
Colgate Total^®^	Regular toothpaste	Water, glycerin, hydrated silica, PVM/MA copolymer, sodium lauryl sulfate, cellulose gum, aroma, sodium hydroxide, carrageenan, sodium fluoride (1450 ppmF^−^), triclosan, sodium saccharin, limonene, CI 77891	Hydrated silica	Colgate-Palmolive Company, Piraeus, Greece

**Table 2:** Means and standard deviations of surface loss (μm) of the experimental groups of the study after tooth-brushing simulation. Surface loss increase (%) in each group compared with negative control group is also presented. The relative dentin abrasivity (RDA) is indicated for each product.

Dentifrice Slurries	Surface Loss (μm)	Surface Loss Increase Compared with Control (−)	RDA	Abrasivity ISO 11609
Deionized water (control −)	6.2 ± 1.5 ^A^	-	5	Low
Instant Whitening Blue Toothpaste	10.4 ± 2.2 ^B^	67.7%	58	Low
Colgate Total	11.0 ± 2.7 ^B^	77.4%	70	Low
Whitening Toothpaste	12.8 ± 2.4 ^C^	106.5%	87	Medium
ISO dentifrice slurry (control +)	14.1 ± 3.3 ^C^	127.4%	100	Medium
Black & Polish Toothpaste	16.6 ± 3.1 ^D^	167.7%	134	High

Same uppercase superscripts in column indicate no significant differences between the groups (*p* > 0.05).

**Table 3:** Means and standard deviations of arithmetical mean height (Sa, μm) of the experimental groups of the study before and after tooth-brushing simulation (TBS). The Sa increase in % of each group is also shown.

Dentifrice Slurries	Sa Before TBS	Sa After TBS	Sa Increase (%) After TBS
Whitening Toothpaste	1.523 ± 0.143 ^Aa^	1.691 ± 0.188 ^Ab^	11.01%
Deionized water (control −)	1.280 ± 0.105 ^Ba^	1.437 ± 0.142 ^Bb^	12.26%
Colgate Total	0.952 ± 0.107 ^Ca^	1.274 ± 0.132 ^Cb^	33.77%
Instant Whitening Blue Toothpaste	1.607 ± 0.265 ^Aa^	2.263 ± 0.361 ^Db^	40.85%
ISO dentifrice slurry (control +)	1.139 ± 0.170 ^Ba^	1.943 ± 0.312 ^Db^	70.59%
Black & Polish Toothpaste	1.608 ± 0.153 ^Aa^	3.950 ± 0.430 ^Eb^	145.64%

Same uppercase superscripts in columns indicate no significant differences among groups (*p* > 0.05). Same lowercase superscripts in rows indicate no significant differences between before and after TBS (*p* > 0.05).

**Table 4:** Means and standard deviations of maximum average between highest peaks and highest valleys of surface (Sz, μm) of the experimental groups of the study before and after tooth-brushing simulation (TBS). The Sz increase in % of each group is also shown.

Dentifrice Slurries	Sz Before TBS	Sz After TBS	SzIncrease (%) After TBS
Deionized water (control −)	8.490 ± 1.811 ^Aa^	9.491 ± 1.832 ^Aa^	11.79 %
Whitening Toothpaste	9.296 ± 1.930 ^Aa^	10.766 ± 2.192 ^Aa^	15.81 %
Colgate Total	8.127 ± 1.406 ^Aa^	10.170 ± 1.723 ^Ab^	25.13 %
Instant Whitening Blue Toothpaste	10.589 ± 2.865 ^Ba^	16.024 ± 3.587 ^Bb^	51.32 %
ISO dentifrice slurry (control +)	9.110 ± 2.256 ^Aa^	15.553 ± 3.821 ^Bb^	70.72 %
Black & Polish Toothpaste	10.100 ± 03.255 ^Ba^	19.658 ± 4.113 ^Cb^	94.63 %

Same uppercase superscripts in columns indicate no significant differences among groups (*p* > 0.05). Same lowercase superscripts in rows indicate no significant differences between before and after TBS (*p* > 0.05).

**Table 5:** Means and standard deviations of developed interfacial area ratio (Sdr, %) of the experimental groups of the study before and after tooth-brushing simulation (TBS). The Sdr increase in % of each group is also shown.

Dentifrice Slurries	Sdr Before TBS	Sdr After TBS	SdrIncrease (%) After TBS
Instant Whitening Blue Toothpaste	5.24 ± 1.65 ^Aa^	6.30 ± 1.91 ^Ab^	20.23%
Colgate Total	5.31 ± 1.48 ^Aa^	6.58 ± 1.72 ^Ab^	23.91%
Deionized water (control −)	5.14 ± 1.11 ^Aa^	6.68 ± 1.41 ^Ab^	29.96%
Whitening Toothpaste	6.27 ± 2.82 ^Aa^	8.28 ± 2.12 ^Ab^	32.06%
ISO dentifrice slurry (control +)	5.48 ± 1.31 ^Aa^	7.30 ± 1.62 ^Ab^	33.21%
Black & Polish Toothpaste	5.59 ± 0.92 ^Aa^	8.91 ± 2.34 ^Bb^	59.39%

Same uppercase superscripts in columns indicate no significant differences among groups (*p* > 0.05). Same lowercase superscripts in rows indicate no significant differences between before and after TBS (*p* > 0.05).

**Table 6:** Means and standard deviations of elemental content (wt%) of dentin surface after the tooth-brushing simulation of each experimental group of the study.

Elements	Deionized Water (− Control)	Colgate Total	Instant Whitening Blue Toothpaste	Whitening Toothpaste	Black & Polish Toothpaste	ISO Dentifrice Slurry (+ Control)
Ca	30.47 ± 4.44 ^a^	30.79 ± 4.02 ^a^	30.34 ± 3.69 ^a^	33.91 ± 4.71 ^a^	30.10 ± 3.77 ^a^	27.95 ± 5.19 ^a^
P	15.64 ± 3.50 ^a^	15.32 ± 2.35 ^a^	16.72 ± 2.81 ^a^	16.57 ± 2.71 ^a^	15.27 ± 2.75 ^a^	14.44 ± 2.49 ^a^
Si	0.00 ± 0.00 ^a^	1.24 ± 0.44 ^b^	1.09 ± 0.54 ^b^	1.20 ± 1.11 ^b^	0.00 ± 0.00 ^a^	0.00 ± 0.00 ^a^
Na	0.00 ± 0.00 ^a^	0.00 ± 0.00 ^a^	0.82 ± 0.16 ^b^	0.00 ± 0.00 ^a^	0.61 ± 0.28 ^b^	0.00 ± 0.00 ^a^
Zn	0.00 ± 0.00 ^a^	0.00 ± 0.00 ^a^	0.00 ± 0.00 ^a^	0.00 ± 0.00 ^a^	0.44 ± 0.18 ^b^	0.00 ± 0.00 ^a^
O	53.88 ± 7.02 ^a^	52.65 ± 5.62 ^a^	52.13 ± 6.46 ^a^	48.32 ± 5.19 ^a^	53.11 ± 7.69 ^a^	57.60 ± 8.32 ^a^

Different lowercase superscripts in rows indicate statistically significant difference (*p <* 0.05).

## Data Availability

The data presented in this study are available upon request from the corresponding author. The data are not publicly available due to privacy.
